# Cannabinoids in Parkinson's Disease

**DOI:** 10.1089/can.2017.0002

**Published:** 2017-02-01

**Authors:** Mario Stampanoni Bassi, Andrea Sancesario, Roberta Morace, Diego Centonze, Ennio Iezzi

**Affiliations:** ^1^ Neurology and Neurorehabilitation Units, IRCCS Istituto Neurologico Mediterraneo (INM) Neuromed, Pozzilli, Italy.; ^2^ Department of Systems Medicine, Tor Vergata University, Rome, Italy.

**Keywords:** basal ganglia, cannabinoids, dopamine, levodopa-induced dyskinesia, Parkinson's disease

## Abstract

The endocannabinoid system plays a regulatory role in a number of physiological processes and has been found altered in different pathological conditions, including movement disorders. The interactions between cannabinoids and dopamine in the basal ganglia are remarkably complex and involve both the modulation of other neurotransmitters (γ-aminobutyric acid, glutamate, opioids, peptides) and the activation of different receptors subtypes (cannabinoid receptor type 1 and 2). In the last years, experimental studies contributed to enrich this scenario reporting interactions between cannabinoids and other receptor systems (transient receptor potential vanilloid type 1 cation channel, adenosine receptors, 5-hydroxytryptamine receptors). The improved knowledge, adding new interpretation on the biochemical interaction between cannabinoids and other signaling pathways, may contribute to develop new pharmacological strategies. A number of preclinical studies in different experimental Parkinson's disease (PD) models demonstrated that modulating the cannabinoid system may be useful to treat some motor symptoms. Despite new cannabinoid-based medicines have been proposed for motor and nonmotor symptoms of PD, so far, results from clinical studies are controversial and inconclusive. Further clinical studies involving larger samples of patients, appropriate molecular targets, and specific clinical outcome measures are needed to clarify the effectiveness of cannabinoid-based therapies.

## Introduction

The endocannabinoid system (ECS) modulates a huge range of physiological functions, including mood, cognition, motor control, feeding behavior, and pain.^1–5^ In recent years, a number of studies explored the role of cannabinoids (CBs) in different pathological conditions.

Approximately 105 CBs have been extracted so far from cannabis.^6^ These phytocannabinoids include Δ9-tetrahydrocannabinol (THC) and cannabidiol (CBD).^7^ Several CB-based medicines are currently approved for clinical indications, including pain, anorexia, spasticity, chemotherapy-induced nausea, and severe refractory epileptogenic encephalopathies of the childhood.^5,8^

The ECS is highly represented in the basal ganglia and has been found altered in several movement disorders, including Parkinson's disease (PD).^9–11^ Preclinical research suggests that modulating CB signaling could improve motor symptoms.^12,13^ Among motor symptoms, levodopa-induced dyskinesias (LIDs) dramatically complicate long-term pharmacological treatment in PD patients. LIDs are thought to arise from pulsatile stimulation of dopamine (DA) receptors with progressive sensitization of DA receptor-associated striatal signaling.^14,15^ So far, despite an increased knowledge of CBs–DA interactions at molecular level, the clinical relevance of CB-based therapies on PD motor symptoms and LIDs has been poorly detailed. The aim of this minireview is to provide an overview of the biochemical interactions between CBs and DA. Furthermore, results from preclinical and clinical studies involving CB-based therapies in PD will be discussed.

## Endocannabinoid System and Dopamine

The ECS is constituted by endocannabinoids (eCBs), biosynthesizing (N-arachidonoyl-phosphatidylethanolamine [NAPE]-specific phospholipase D and diacylglycerol [DAG] lipase-a) and degrading (fatty acid amide hydrolysis [FAAH] and monoacylglycerol lipase [MAGL]) enzymes, and CB receptors (CBRs).

The best characterized eCBs (N-arachidonoylethanolamine [AEA] or anandamide and 2-arachidonoylglycerol [2-AG]) interact with the two main CBRs subtypes (CB1R and CB2R) and also with other receptors, including the transient receptor potential vanilloid type 1 (TRPV1) cation channel,^16^ the GTP-binding protein-coupled receptor GPR55,^17^ the abnormal-CBD receptor,^18^ and the peroxisome proliferator-activated receptor (PPAR).^19^

eCBs regulate synaptic transmission producing a physiological feedback mechanism aimed to prevent an excess of excitation or inhibition.^20^ This “retrograde signaling”^21^ results in depolarization-induced suppression of inhibition (DSI) at γ-aminobutyric acid (GABA)ergic synapses and in depolarization-induced suppression of excitation (DSE) at glutamatergic synapses.^22–24^ The presynaptic location of CB1R, also allows eCBs to directly modulate other neurotransmitters, including opioid peptides, acetylcholine, and 5-hydroxytryptamine (5-HT).^25,26^

Although nigrostriatal dopaminergic neurons seem not to express CB1R,^27,28^ they are significantly affected by either the activation or the blockade of the ECS.^29,30^ These effects are likely mediated by CB1R located in other neuronal subpopulations (i.e., GABAergic, glutamatergic, and opioidergic neurons) located near to and connected with dopaminergic neurons.^10,31,32^ Indeed, it should be reminded that dopaminergic neurons may, in turn, produce eCBs from their somata and dendrites,^33,34^ thus facilitating the retrograde signaling at excitatory and inhibitory synapses.^35^

Additional direct mechanisms have been proposed to explain the modulation of eCBs on DA transmission. Some eCBs, including AEA, have been found to interact with TRPV1 receptors,^36^ which are expressed in dopaminergic neurons.^37^ CB1R can form heteromers with other metabotropic receptors, including the dopamine D1 and D2 receptor.^38^ Finally, CB2R have been identified in human nigrostriatal dopaminergic neurons,^12^ this may support a direct role of eCBs in modulating dopaminergic transmission.

## CB–DA Interactions in the Basal Ganglia

Activation of the ECS has been associated with motor inhibition and reduced dopaminergic activity. Classically, in hyperkinetic conditions reduced eCB tone accompany increased dopaminergic activity, whereas in hypokinetic movement disorders, the opposite pattern is observed.^29^ In experimental models of PD, eCBs can enhance the hypokinetic effects of DA-depleting agents and reduce the effects of drugs producing hyperstimulation of DA receptors.^27,29^ In particular, it has been proposed that motor inhibition produced by CB1R stimulation is mediated by the regulation of the phosphorylation state of a critical mediator of DA action in striatal neurons, DA- and cAMP-regulated phosphoprotein of 32 kDa^39^ (DARPP-32).

At cellular level, CB–DA interactions seem to be much more complex. First, dopaminergic transmission can influence the eCBs levels in the striatum as shown by the increase of AEA levels after D2-like receptor stimulation.^40,41^ This effect depends on both stimulation of its synthesis and inhibition of its degradation, as suggested by the ability of D2-like receptor agonists to modulate the activity of NAPE-phospholipase D and FAAH. Such DA-stimulated eCB activity can counter the action of D2 receptor activation in the striatum, suggesting an inhibitory feedback mechanism aimed at limiting the hyperkinetic effect of DA. To add more complexity, a cooperative action of CB1 and D2 receptors has also been proposed by the findings that AEA produced by DA stimulation can enhance the effects of D2 receptor activation.^42–44^ Indeed, inhibition of GABA transmission via D2-like receptors can be partly prevented by CBR blockade suggesting that eCBs may act as downstream effectors of D2 receptors.^45^ Accordingly, both D2 and CB1 receptors are expressed on GABA terminals of the striatum.^46–48^

**FIG. 1.**
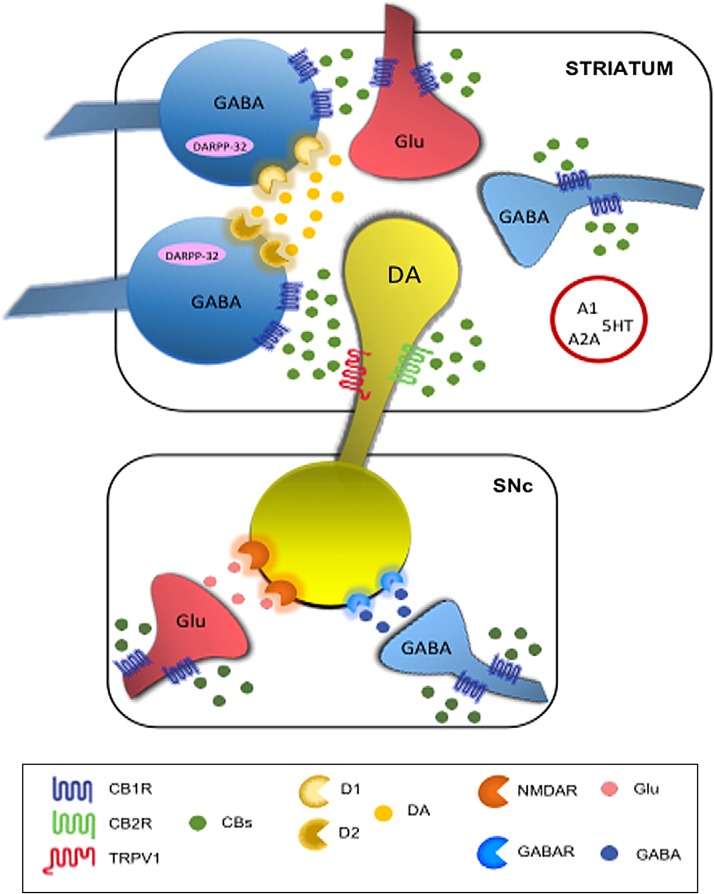
Schematic mechanisms explaining the interactions between cannabinoid system and dopaminergic transmission at basal ganglia level. In the red circle are depicted additional receptors involved in cannabinoid signaling. A1, adenosine A1 receptor; A2A, adenosine A2A receptor; CB1R, cannabinoid receptor type 1; CB2R, cannabinoid receptor type 1; CBs, cannabinoids; D1, dopamine receptor type 1; D2, dopamine receptor type 2; DA, dopamine; DARPP-32, DA- and cAMP regulated phosphoprotein of 32 kDa; Glu, glutamate; GABAR, γ-aminobutyric acid receptor; GABA, γ-aminobutyric acid; 5HT, 5-hydroxytryptamine receptor; NMDAR, N-methyl-d-aspartate receptor; TRPV1, transient receptor vanilloid type 1 cation channel.

The complex interaction between DA and eCBs (Fig. 1) well explains the reorganization of these systems in both idiopathic and experimental PD. Previous studies in experimental PD showed enhanced eCB activity in the basal ganglia, including increased CB1 mRNA levels, CB1 activity, AEA levels, and decreased CB clearance.^9,10,27,49–52^ Accordingly, increased level of AEA has been shown in the cerebrospinal fluid of untreated PD patients.^11^ Also, increased expression of CB1 receptors in the basal ganglia has been reported.^51^ These changes are associated with movement suppression and may be reversed by chronic levodopa treatment.^9,51,53^

Whereas some of these alterations may reflect endogenous compensatory mechanisms aimed at limiting the effects of DA loss in the basal ganglia, others probably contribute in generating the typical parkinsonian motor symptoms.^52^

## Basal Ganglia Plasticity in LID

**FIG. 2.**
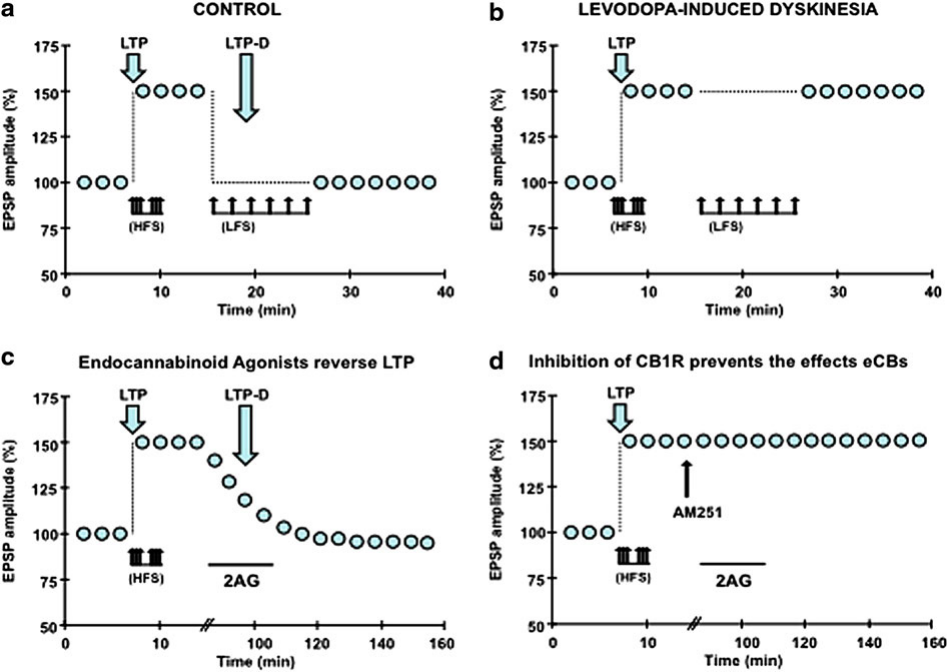
Synaptic plasticity in levodopa-induced dyskinesia and role of endocannabinoids in synaptic depotentiation. **(a)** In normal conditions, HFS induces LTP of the amplitude of EPSPs. LFS delivered after LTP induction reset synapses to naïve state. **(b)** In levodopa-induced dyskinesia, HFS produced LTP as in control condition, but LFS failed to induce LTP-D. **(c)** Perfusion of 20 μM 2AG (black bar), an endocannabinoid agonist, reversed LTP induced by HFS. **(d)** The effects of 2AG on LTP were blocked by 5 μM AM251, an inhibitor of CB1 receptors. 2AG, 2-arachidonoylglycerol; EPSP, excitatory postsynaptic potential; HFS, high-frequency stimulation; LFS, low-frequency stimulation; LTP, long-term potentiation; LTP-D, depotentiation.

DA plays a pivotal role in producing two opposite forms of corticostriatal synaptic plasticity: long-term depression (LTD) and long-term potentiation (LTP). LTD makes glutamatergic synapses less excitable to future stimulation, LTP strengthens the connections between cortical and striatal neurons (Fig. 2a). The reversal of LTP is termed depotentiation (LTP-D) and operates to reset synaptic transmission to the naive state.^54,55^ Although both depotentiation and LTD reduce the strength of synaptic transmission, depotentiation is unable *per se* to depress nonpotentiated synapses^15^ and requires N-methyl-d-aspartate (NMDA) receptors activation.^15,56^ In experimental PD, LIDs are associated with aberrant corticostriatal plasticity (Fig. 2b), in particular, corticostriatal LTP is favored over LTD^15,56^ and is also abnormally stable and refractory to depotentiation.^57^

Depotentiation can follow different mechanism, homosynaptic LTP-D requiring the activation of the same pathways that triggered LTP^58,59^; conversely, heterosynaptic LTP-D involves inputs different from those engaged in LTP. Previous studies have shown that heterosynaptic LTP-D entails CB1, GABA-A, and adenosine A1 receptors, and ERK 1/2 and p38 MAPK signaling and also showed that eCBs play a complex role in both presynaptic and postsynaptic changes^60^ (Fig. 2c, d). It is worth noting that activation of adenosine A1 receptors is also involved in other forms of LTD and depotentiation.^61–64^

## Preclinical Studies

Preclinical studies using different models of experimental PD have investigated the effects of both agonists and antagonists of the CBR, used alone or as coadjuvants.^13,29,52,65^

CB1 agonists inhibit basal ganglia DA release and are therefore expected to be ineffective in alleviating PD motor symptoms. CB1 agonists exacerbated bradykinesia in 1-methyl-4-phenyl-1,2,3,6-tetrahydropyridine (MPTP)-lesioned primates.^66^ However, different CB1 agonists have also been reported to improve motor impairment, possibly through nondopaminergic mechanisms, including interactions with adenosine A2A and 5-HT receptors.^67–72^

Studies of CB1 antagonists more consistently showed improvement of motor symptoms.^73–77^ Blockade of CB1R with rimonabant or other antagonists reduced akinesia and motor impairment in experimental models of PD,^73,75,77,78^ although a few other studies showed conflicting results.^9,66^ Moreover, rimonabant was more effective when used at low doses,^75,77^ and in very advanced phases of the disease characterized by extreme nigral damage.^73^ These effects appear to involve nondopaminergic mechanisms, including enhanced striatal glutamate release.^9,73,75^

The ECS might be involved in LIDs, although the results are controversial. Although this system is modulated in different experimental models of PD and in response to chronic levodopa treatment,^51,79^ it is not known whether these changes are compensatory or causal.^80^ Preclinical studies showed that both CB1R agonists and antagonists represent potentially useful antidyskinetic agents.^69,74,81^

The antidyskinetic effects of CBR agonists^81–84^ are mediated by a normalization of cAMP/PKA signaling and are associated to an increased DARPP-32 phosphorylation.^84^ However, as higher doses of CB1 agonists may impair motor function, it has been suggested that the effects on LIDs may be related to a global motor inhibition.^85^ In one study, FAAH inhibitors failed to reproduce the beneficial effects of CB agonists when given alone. As FAAH inhibitors showed antidyskinetic properties only when combined with a TRPV1 receptor antagonist, it is conceivable that CB1 and TRPV1 receptors operate in opposite directions to control LIDs.^83^ A recent study added more complexity by suggesting that certain CBs (e.g., AEA) may reduce LIDs by activating PPAR-γ.^86^ Beneficial effects were also reported for the PPAR-α receptor endogenous lipid ligand oleoylethanolamide, although the antidyskinetic effect was attributed to the blockade of TRPV1 receptors rather than the activation of PPAR-α receptors.^87^

## Clinical Studies

Observational studies suggest that CBs may improve some motor and nonmotor symptoms associated to PD (Table 1). In two published surveys of PD patients, smoked cannabis was reported to produce some benefit on motor and nonmotor symptoms, although these studies present several limitations that could have influenced the results.^88,89^ A small case series showed no benefit for tremor following a single administration of smoked cannabis.^90^ In contrast, a small open-label study assessing motor exam 30 min after smoking cannabis reported improvement in tremor, rigidity, bradykinesia, pain, and sleep.^91^ Regarding nonmotor symptoms, a small 4-week open-label study of CBD for psychosis in PD found improvement on the Brief Psychiatric Rating Scale and Parkinson Psychosis Questionnaire,^92^ and another small case series reported benefits for rapid eye movement sleep behavior disorder.^93^

**Table 1.** **Clinical Studies Examining Whether Cannabinoids Improve Motor and Nonmotor Symptoms in Parkinson's Disease**

Study design	Number of patients	Cannabinoids	Results	Authors
Patient survey	84	Smoked cannabis	Forty-six percent of patients described some benefit; 31% reported improvement of rest tremor, 45% of bradykinesia and 14% of LID	Venderová et al.^88^
Patient survey	9	Cannabis	Seven patients (78%) reported improvement of mood and sleep, two patients reported improved motor symptoms, not specifically dyskinesias	Finseth et al.^89^
Case series	5	Smoked cannabis, 1 g cannabis (2–9% THC)	No benefit for tremor following single administration	Frankel et al.^90^
Open-label	22	Smoked cannabis, 0.5 g cannabis	Thirty minutes after smoking cannabis, patients reported improvement in tremor, rigidity, bradykinesia, pain, and sleep	Lotan et al.^91^
Four-week open-label	6	CBD up to 400 mg/day	Improvements on the Brief Psychiatric Rating Scale and Parkinson Psychosis Questionnaire	Zuardi et al.^92^
Case series	4	CBD 75 or 300 mg/day	Benefits for rapid eye movement sleep behavior disorder	Chagas et al.^93^
Randomized, double-blind, placebo-controlled crossover	5	Nabilone	Significant reduction of the Rush Dyskinesia Disability Scale and total LID time; two patients reported improvement in painful off-dystonia	Sieradzan et al.^94^
Four-week randomized, double-blind, placebo-controlled crossover	17	Cannador (1.25 mg CBD and 2.5 mg THC)	No improvement of LIDs on multiple outcomes.	Carroll et al.^95^
			No significant changes for motor symptoms (UPDRS-III), quality of life (PDQ-39) or sleep	
Randomized, double-blind, placebo-controlled	8	Rimonabant	No effect on motor symptoms or LID (UPDRS and standardized videotape)	Mesnage et al.^96^
Randomized, double-blind, placebo-controlled	21	CBD 75 or 300 mg/day	No changes for total UPDRS or any subscales.	Chagas et al.^97^
			Improvement for total PDQ-39 score and activities of daily living subscores for the CBD 300 mg/day group	

CBD, cannabidiol; LID, levodopa-induced dyskinesia; PDQ-39, Parkinson's Disease Questionnaire-39; THC, tetrahydrocannabinol; UPDRS, Unified Parkinson's Disease Rating Scale.

Few controlled clinical studies explored the effects of CBs on motor and nonmotor symptoms in PD patients.^94–97^ A small randomized, double-blind, placebo-controlled crossover trial (Class III) assessing the efficacy on LIDs of nabilone (CB1 and CB2 agonist) showed reduction of the Rush Dyskinesia Disability Scale and of total LID time.^94^ A small 4-week randomized double-blind crossover study (Class I) explored the effect of Cannador (oral cannabis extract: 1.25 mg CBD and 2.5 mg THC) on LIDs.^95^ Cannador failed to improve LIDs. Moreover, no significant changes were observed for other secondary outcomes, including motor symptoms (Unified Parkinson's Disease Rating Scale [UPDRS-III]), quality of life (Parkinson's Disease Questionnaire-39 [PDQ-39]), or sleep. However, it should be again considered that some issues compromised the results (i.e., 71% correct identification of treatment). Most recently, 21 PD patients were randomized to placebo, CBD 75 mg/day, or CBD 300 mg/day for a 6-week trial.^97^ Although no significant changes were found for the total UPDRS, some improvement was noted in the CBD 300 mg/day group for the quality of life (total PDQ-39 score and activities of daily living subscores).

For the purposes of this minireview, it should be mentioned another small 16-day randomized placebo-controlled trial assessing the efficacy of 20 mg daily oral rimonabant (CB1 antagonist)s, which showed no effect on parkinsonian motor symptoms or LIDs as measured by the UPDRS and a standardized videotape procedure.^96^

Despite the low sample size and quality of these studies, the data suggest that some motor symptoms in PD, in particular LIDs, may respond to cannabis-based therapies.^98^ Indeed, several factors (i.e., disease stage and levodopa treatment, lack of standardized methods) may explain the conflicting findings. While no serious adverse events were reported, side effects included hypotension, vertigo, visual hallucinations, dizziness, and somnolence. Further studies are warranted using different doses, formulations or target symptoms (e.g., dystonia, psychosis, sleep).

## Conclusions

Cannabis is a psychoactive compound widely used along history for recreational and therapeutic purposes. Although many open questions remain, cannabis-based therapies have become increasingly common raising considerable interest in politics as well as in general public for legalization of medical cannabis.

In recent years, a growing body of literature addressed the role of CBs in physiological and pathological conditions. In movement disorders, preclinical studies strongly contributed to increase knowledge on the interaction between CBs, DA, and other signaling pathways, adding novel insight on pathophysiology and contributing to identify new pharmacological targets.

Results from available clinical studies are controversial and inconclusive due to several limitations, including small sample size, lack of standardized outcome measures, and expectancy bias. Well-designed studies involving larger sample of patients, appropriate molecular targets, objective biological measures (i.e., CBs blood level), and specific clinical outcome measures are needed to clarify the effectiveness of CB-based therapies. In addition, health concerns associated with medical cannabis use have to be carefully addressed by preclinical safety studies evaluating acute and long-term effects on motor functions as well on mood and cognition.

In this view, ongoing research and public policy should help to clarify these issues reducing the incongruence between approved and actual use of medical cannabis.

## Abbreviations Used

AEA: N-arachidonoylethanolamine
2-AG: 2-arachidonoylglycerol
CB: cannabinoid
CBD: cannabidiol
CBRs: CB receptors
DA: dopamine
DARPP-32: DA- and cAMP-regulated phosphoprotein of 32 kDa
DAG: diacylglycerol
DSE: depolarization-induced suppression of excitation
DSI: depolarization-induced suppression of inhibition
eCBs: endocannabinoids
ECS: endocannabinoid system
FAAH: fatty acid amide hydrolysis
GABA: γ-aminobutyric acid
5-HT: 5-hydroxytryptamine
LIDs: levodopa-induced dyskinesias
LTD: long-term depression
LTP: long-term potentiation
LTP-D: depotentiation
MAGL: monoacylglycerol lipase
MPTP: 1-methyl-4-phenyl-1,2,3,6-tetrahydropyridine
NAPE: N-arachidonoyl-phosphatidylethanolamine
NMDA: N-methyl-D-aspartate
PD: Parkinson's disease
PPAR: peroxisome-proliferator-activated receptor
THC: tetrahydrocannabinol
TRPV1: transient receptor potential vanilloid type 1
